# Supplementary material for: Resolving the full spectrum of human genome variation using Linked-Reads
Source: Genome Res. 2019 Apr;29(4):635–45. doi: 10.1101/gr.234443.118 (PMC6442396; doi:10.1101/gr.234443.118)
Supplement: Supplemental Material [file supp_gr.234443.118_Supplemental_longranger-2.2.zip › longranger-2.2.tar.bz2!/lr2.2-public/lib/stan/cmdstan/stan/doc/api/html/internal__math_8hpp_source.html]

Stan: src/stan/prob/internal\_math.hpp Source File


|  |  |
| --- | --- |
|  | Stan  2.5.0  probability, sampling & optimization |


- Main Page
- Related Pages
- Namespaces
- Classes
- Files

- File List
- File Members

All Classes Namespaces Files Functions Variables Typedefs Enumerations Enumerator Friends Macros Pages

- src
- stan
- prob

internal\_math.hpp

Go to the documentation of this file.

1 #ifndef STAN\_\_PROB\_\_INTERNAL\_MATH\_HPP

2 #define STAN\_\_PROB\_\_INTERNAL\_MATH\_HPP

3

4 #include <math.h>

5 #include <boost/math/special\_functions/gamma.hpp>

6 #include <boost/math/special\_functions/beta.hpp>

7 #include <boost/math/special\_functions/fpclassify.hpp>

8

9 namespace stan {

10

11  namespace math {

12

13

14  double F32(double a, double b, double c, double d, double e, double z, double precision = 1e-6)

15  {

16

17  double F = 1;

18

19  double tNew = 0;

20

21  double logT = 0;

22

23  double logZ = std::log(z);

24

25  int k = 0;

26

27  while( (fabs(tNew) > precision) || (k == 0) )

28  {

29

30  double p = (a + k) \* (b + k) \* (c + k) / ( (d + k) \* (e + k) \* (k + 1) );

31

32  // If a, b, or c is a negative integer then the series terminates

33  // after a finite number of interations

34  if(p == 0) break;

35

36  logT += (p > 0 ? 1 : -1) \* std::log(fabs(p)) + logZ;

37

38  tNew = std::exp(logT);

39

40  F += tNew;

41

42  ++k;

43

44  }

45

46  return F;

47

48  }

49

50  void gradF32(double\* g, double a, double b, double c, double d, double e, double z, double precision = 1e-6)

51  {

52

53  double gOld[6];

54

55  for(double \*p = g; p != g + 6; ++p) \*p = 0;

56  for(double \*p = gOld; p != gOld + 6; ++p) \*p = 0;

57

58  double tOld = 1;

59  double tNew = 0;

60

61  double logT = 0;

62

63  double logZ = std::log(z);

64

65  int k = 0;

66

67  while( (fabs(tNew) > precision) || (k == 0) )

68  {

69

70  double C = (a + k) / (d + k);

71  C \*= (b + k) / (e + k);

72  C \*= (c + k) / (1 + k);

73

74  // If a, b, or c is a negative integer then the series terminates

75  // after a finite number of interations

76  if(C == 0) break;

77

78  logT += (C > 0 ? 1 : -1) \* std::log(fabs(C)) + logZ;

79

80  tNew = std::exp(logT);

81

82  gOld[0] = tNew \* (gOld[0] / tOld + 1.0 / (a + k) );

83  gOld[1] = tNew \* (gOld[1] / tOld + 1.0 / (b + k) );

84  gOld[2] = tNew \* (gOld[2] / tOld + 1.0 / (c + k) );

85

86  gOld[3] = tNew \* (gOld[3] / tOld - 1.0 / (d + k) );

87  gOld[4] = tNew \* (gOld[4] / tOld - 1.0 / (e + k) );

88

89  gOld[5] = tNew \* ( gOld[5] / tOld + 1.0 / z );

90

91  for(int i = 0; i < 6; ++i) g[i] += gOld[i];

92

93  tOld = tNew;

94

95  ++k;

96

97  }

98

99  }

100

101  // Gradient of the hypergeometric function 2F1(a, b | c | z) with respect to a and c

102  void grad2F1(double& gradA, double& gradC, double a, double b, double c, double z, double precision = 1e-6)

103  {

104

105  gradA = 0;

106  gradC = 0;

107

108  double gradAold = 0;

109  double gradCold = 0;

110

111  int k = 0;

112  double tDak = 1.0 / (a - 1);

113

114  while( (fabs(tDak \* (a + (k - 1)) ) > precision) || (k == 0) )

115  {

116

117  const double r = ( (a + k) / (c + k) ) \* ( (b + k) / (double)(k + 1) ) \* z;

118  tDak = r \* tDak \* (a + (k - 1)) / (a + k);

119

120  if(r == 0) break;

121

122  gradAold = r \* gradAold + tDak;

123  gradCold = r \* gradCold - tDak \* ((a + k) / (c + k));

124

125  gradA += gradAold;

126  gradC += gradCold;

127

128  ++k;

129

130  if(k > 200) break;

131

132  }

133

134  }

135

136  // Gradient of the incomplete beta function beta(a, b, z)

137  // with respect to the first two arguments, using the

138  // equivalence to a hypergeometric function.

139  // See http://dlmf.nist.gov/8.17#ii

140  void gradIncBeta(double& g1, double& g2, double a, double b, double z)

141  {

142

143  double c1 = std::log(z);

144  double c2 = std::log(1 - z);

145  double c3 = boost::math::beta(a, b, z);

146

147  double C = std::exp( a \* c1 + b \* c2 ) / a;

148

149  double dF1 = 0;

150  double dF2 = 0;

151

152  if(C) grad2F1(dF1, dF2, a + b, 1, a + 1, z);

153

154

155  g1 = (c1 - 1.0 / a) \* c3 + C \* (dF1 + dF2);

156  g2 = c2 \* c3 + C \* dF1;

157

158  }

159

160  // Gradient of the regularized incomplete beta function ibeta(a, b, z)

161  void gradRegIncBeta(double& g1, double& g2, double a, double b, double z,

162  double digammaA, double digammaB, double digammaSum, double betaAB)

163  {

164

165  double dBda = 0;

166  double dBdb = 0;

167

168  gradIncBeta(dBda, dBdb, a, b, z);

169

170  double b1 = boost::math::beta(a, b, z);

171

172  g1 = ( dBda - b1 \* (digammaA - digammaSum) ) / betaAB;

173  g2 = ( dBdb - b1 \* (digammaB - digammaSum) ) / betaAB;

174

175  }

176

177  // Gradient of the regularized incomplete gamma functions igamma(a, g)

178  // Precomputed values

179  // g = boost::math::tgamma(a)

180  // dig = boost::math::digamma(a)

181  double gradRegIncGamma(double a, double z, double g, double dig,

182  double precision = 1e-6) {

183  using boost::math::gamma\_p;

184

185  double S = 0;

186  double s = 1;

187  double l = std::log(z);

188

189  int k = 0;

190  double delta = s / (a \* a);

191

192  while (fabs(delta) > precision) {

193  S += delta;

194  ++k;

195  s \*= - z / k;

196  delta = s / ((k + a) \* (k + a));

197  if (boost::math::isinf(delta))

198  throw std::domain\_error("stan::math::gradRegIncGamma not converging");

199  }

200  return gamma\_p(a, z) \* ( dig - l ) + std::exp( a \* l ) \* S / g;

201  }

202

203  }

204

205 }

206

207 #endif

stan::math::grad2F1

void grad2F1(double &gradA, double &gradC, double a, double b, double c, double z, double precision=1e-6)

**Definition:** internal\_math.hpp:102

stan::agrad::fabs

fvar< T > fabs(const fvar< T > &x)

**Definition:** fabs.hpp:18

stan::math::gradRegIncBeta

void gradRegIncBeta(double &g1, double &g2, double a, double b, double z, double digammaA, double digammaB, double digammaSum, double betaAB)

**Definition:** internal\_math.hpp:161

boost::math::isinf

bool isinf(const stan::agrad::var &v)

Checks if the given number is infinite.

**Definition:** boost\_fpclassify.hpp:54

stan::math::gradF32

void gradF32(double \*g, double a, double b, double c, double d, double e, double z, double precision=1e-6)

**Definition:** internal\_math.hpp:50

stan::math::gradRegIncGamma

double gradRegIncGamma(double a, double z, double g, double dig, double precision=1e-6)

**Definition:** internal\_math.hpp:181

stan::math::F32

double F32(double a, double b, double c, double d, double e, double z, double precision=1e-6)

**Definition:** internal\_math.hpp:14

stan::agrad::gamma\_p

fvar< T > gamma\_p(const fvar< T > &x1, const fvar< T > &x2)

**Definition:** gamma\_p.hpp:16

stan::math::gradIncBeta

void gradIncBeta(double &g1, double &g2, double a, double b, double z)

**Definition:** internal\_math.hpp:140

stan::math::e

double e()

Return the base of the natural logarithm.

**Definition:** constants.hpp:86

stan::agrad::log

fvar< T > log(const fvar< T > &x)

**Definition:** log.hpp:15

stan::agrad::exp

fvar< T > exp(const fvar< T > &x)

**Definition:** exp.hpp:16

stan::math::gamma\_p

double gamma\_p(double x, double a)

**Definition:** gamma\_p.hpp:53


---

[ Stan Home Page ]

*© 2011–2014,
Stan Development Team.*
